# Perceptions of Stroke and Associated Health-Care-Seeking Behavior in Northern Tanzania: A Community-Based Study

**DOI:** 10.1159/000499069

**Published:** 2019-04-15

**Authors:** Julian T. Hertz, Deng B. Madut, Gwamaka William, Venance P. Maro, John A. Crump, Matthew P. Rubach

**Affiliations:** a Division of Emergency Medicine, Duke University Medical Center, Durham, NC, USA;; b Department of Medicine, Duke University Medical Center, Durham, NC, USA;; c Kilimanjaro Christian Medical Centre, Moshi, Tanzania;; d Department of Medicine, Kilimanjaro Christian Medical Centre, Moshi, Tanzania;; e Otago Global Health Institute, University of Otago, Dunedin, New Zealand

**Keywords:** Stroke, Sub-Saharan Africa, Knowledge, Health-care-seeking behavior, Tanzania

## Abstract

**Background:**

Little is known about knowledge of stroke symptoms, perceptions of self-risk, and health-care-seeking behavior for stroke in East Africa.

**Methods:**

A 2-stage randomized population-based cluster survey with selection proportional to population size was performed in northern Tanzania. Self-identified household health-care decision makers were asked to list all symptoms of a stroke. They were further asked if they thought they had a chance of having a stroke and where they would present for care for stroke-like symptoms. A socioeconomic status score was derived via principal component analysis from 9 variables related to wealth.

**Results:**

Of 670 respondents, 184 (27.4%) knew a conventional stroke symptom and 51 (7.6%) thought they had a chance of having a stroke. Females were less likely to perceive themselves to be at risk than males (OR 0.49, 95% CI 0.28–0.89, *p* = 0.014). Of respondents, 558 (88.3%) stated they would present to a hospital for stroke-like symptoms. Preference for a hospital was not associated with knowledge of stroke symptoms or perception of self-risk but was associated with a higher socioeconomic status score (*p* < 0.001).

**Conclusions:**

Knowledge of stroke symptoms and perception of self-risk are low in northern Tanzania, but most residents would present to a hospital for stroke-like symptoms.

## Introduction

Stroke is a life-threatening emergency and a leading cause of death and disability worldwide [1]. Stroke disproportionately affects persons living in low- and middle-income countries, where the majority of stroke-related disability and stroke mortality occur [2]. In sub-Saharan Africa (SSA), the burden of stroke is substantial and growing [3–6], and limited existing data suggest that the incidence of stroke in many parts of SSA is higher than in high-income countries [2]. The estimated incidence of stroke in Tanzania, for example, is among the highest in the world with an annual 109–316 strokes per 100,000 persons [7, 8].

Given the growing burden of disease, there has been increasing study of community knowledge of stroke symptoms in SSA. Such research has been conducted mostly in West Africa, where between 33 and 98% of respondents could identify at least one stroke symptom [9–16]. Very little study has been done of stroke symptom awareness in East Africa, despite evidence that the incidence of stroke is higher in East than West Africa [2, 7]. The only existing data regarding knowledge of stroke symptoms in East Africa come from Uganda, where 25–43% of participants knew a stroke symptom [17, 18]. In Tanzania, to our knowledge, there has been no study to date of community awareness of stroke symptoms.

Little is known about health-care-seeking behavior for stroke and perception of self-risk of stroke in SSA. In Ghana, Nigeria, and Uganda, between 61 and 85% of respondents reported that they would present to a hospital if they thought they were having a stroke [10, 11, 17], but there are no other data about patterns of care seeking for stroke across SSA. With regard to perception of self-risk, only 66% of participants in a single study in Uganda thought they were at risk of suffering a stroke [17], but to our knowledge, no other study of perceptions of self-risk has been done in SSA.

Developing a greater understanding of stroke symptom knowledge, perception of self-risk, and health-care-seeking behavior in SSA is needed to identify knowledge gaps, describe current patterns of care seeking, and develop educational interventions regarding stroke. In high-income settings, earlier presentation to hospital and earlier treatment of stroke result in better long-term neurologic outcomes [19], but it is unknown if prompt hospital presentation for stroke improves morbidity or mortality in SSA. Thus, understanding patterns of care seeking for stroke in SSA and the way such behavior is influenced by stroke symptom knowledge and perception of self-risk may be important in designing educational programs to stem the rising tide of stroke-related mortality and disability across SSA.

We sought to describe knowledge of stroke symptoms, perception of self-risk, health-care-seeking behavior for stroke-like symptoms, and the interaction between these factors in Tanzania. To do so, we conducted a community-based survey of adults in an area of Tanzania where the burden of stroke is high and growing [8, 20].

## Materials and Methods

### Study Location

This study was done in northern Tanzania in the city of Moshi (population 184,289 [21]) and the surrounding rural districts of Hai (population 210,531 [21]) and Moshi Rural (population 466,740 [21]). In 2014, the prevalence of hypertension among adults in Moshi Urban and Moshi Rural districts was 28%, and the prevalence of diabetes was 6% [22, 23].

### Participant Selection

A 2-stage randomized population-based cluster survey was performed, following World Health Organization recommendations for vaccination coverage cluster surveys [24]. Within the study area, 56 subdistricts were selected randomly in a population-weighted fashion, with selection of urban and rural subdistricts proportionate to their populations. Within each selected subdistrict, 12 point locations were generated randomly using Quantum Geographic Information System (version 2.18.7). The global positioning system coordinates of each selected point were entered into a Garmin eTrex handheld device (Garmin, Olathe, KS, USA), which was used to locate the physical location of each random point. The closest household to the selected point was approached for inclusion in the study; if there was no eligible participant at this household, then the next nearest household was approached. Any adult resident who self-identified as a health-care decision maker for the household was eligible for inclusion.

### Survey Translation

Survey questions were translated into Swahili and back-translated into English. To ensure proper translation of the word “stroke,” we piloted several word choices with 15 Tanzanians with both medical and nonmedical backgrounds, who were in unanimous agreement that the appropriate term in Swahili is “*kiharusi*” but that the English word “stroke” is more widely used and recognized. For this reason, both “*kiharusi*” and “stroke” were used in all questions regarding stroke to optimize comprehension.

### Study Procedures

Surveys were administered in Swahili by Tanzanian field workers, using Samsung Galaxy Tab A tablets (Samsung, Seoul, Korea). Surveys were designed using Open Data Kit software version 1.12.2 (Seattle, WA, USA). Basic sociodemographic information was collected from all respondents, including age and gender, as well as education of the head of household. Participants were asked to list as many symptoms of a stroke as they could think of. Surveyors did not present participants with a list of options; participants had to name symptoms without prompting. When participants struggled to understand the biomedical concept of “symptoms,” surveyors explained this concept but ultimately recorded the participant’s response exactly as given. Respondents were subsequently asked “Do you think you have a chance of having a stroke?” Finally, respondents were asked where they would go if they or another adult in their household were to experience a standardized stroke scenario of sudden unilateral paralysis, trouble speaking, trouble walking, confusion, or vision problems. The scenario was adapted from US National Institute for Neurologic Disorders and Stroke (US NINDS) guidelines [25]. This standardized stroke scenario was piloted with local clinicians to ensure that it adequately conveyed a stroke presentation in Tanzania. As a result of this piloting, some symptoms in the US NINDS scenario, such as dizziness and loss of balance or coordination, were eliminated due to concerns that the best Swahili translation of these words would not adequately convey the intended symptom and that “trouble walking” would better convey deficits in balance and coordination in Swahili. In response to the standardized stroke scenario, participants were asked to choose from a pick list of common types of health-care facilities in Tanzania as well as self-treatment at home and traditional healer. The full survey instrument, in both English and Swahili, is provided in the online supplementary material (for all online suppl. material, see www.karger.com/doi/10.1159/000499069).

### Statistical Analyses

Statistical analyses were performed in RStudio version 3.3.2 (RStudio Inc., Boston, MA, USA). Associations between categorical variables were analyzed with Pearson’s chi-square, and continuous variables were analyzed using the Welch 2 sample *t* test. ORs and corresponding CIs were calculated from contingency tables. A 0.05 cutoff level for statistical significance was used. Urban residence was defined as residence within Moshi Urban District. Conventional stroke symptoms were defined a priori as focal weakness, focal numbness, headache, dizziness, trouble seeing, confusion, or trouble speaking, consistent with the US NINDS guidelines [25]. A socioeconomic status score was derived via principal component analysis [26] from 9 binary variables: post-primary education, presence of electricity in the home, health insurance coverage, home floor material, ownership of a bank account, ownership of a car, ownership of a television, ownership of a refrigerator, and presence of a flush toilet in the home. The minimum sample size was calculated according to WHO guidelines for cluster surveys [24], assuming a proportion (defined as the study area’s population that sought health care at a specific health-care facility) of 0.1, a precision of 10%, a design effect of 1.5, and an inflation factor of 1.2. The sample size calculation resulted in a minimum of 660 households to be surveyed. Subdistrict population data were taken from the 2012 Tanzania Population and Housing Census [21].

## Results

There were 670 participants, with median (range) age 47 (16–98) years. Of participants, 469 (70.0%) were females. Table 1 presents the sociodemographic features of survey respondents.

### Knowledge of Stroke Symptoms

The symptoms of stroke identified by participants are summarized in Table 2. The most commonly identified stroke symptom was focal weakness, cited by 135 (20.1%) respondents. Of participants, 184 (27.4%) were able to identify at least one conventional stroke symptom, 33 (4.9%) were able to identify at least 2 conventional symptoms, and 3 (0.4%) were able to identify at least 3 conventional symptoms. The least frequently mentioned conventional stroke symptom was trouble seeing, which was cited by 1 (0.1%) respondent. There was no significant difference in age among those who could identify a conventional stroke symptom versus those who could not (mean age 48.1 vs. 48.0 years, *p* = 0.957).

### Perception of Stroke Risk

Of respondents, 51 (7.6%) thought they had a chance of having a stroke, whereas 311 (46.4%) thought they had no chance of having a stroke and 308 (46.0%) were unsure. Those who perceived themselves to be at risk of a stroke were not significantly older than other respondents (mean age 52.0 vs. 47.7 years, *p* = 0.082). Females were significantly less likely to perceive themselves to be at risk of stroke than males (6.0 vs. 11.4%, OR 0.49, 95% CI 0.28–0.89, *p* = 0.014).

### Health-Care-Seeking Behavior for Stroke Symptoms

The responses to the question “Where would you go if you or another adult in your household experienced sudden unilateral paralysis, trouble speaking, trouble walking, confusion, or trouble seeing?” are summarized in Table 3. The most commonly selected facility was a hospital, chosen by 558 (88.3%) of participants. No participant stated they would present to a clinic or a pharmacy.

Table 4 compares the characteristics of respondents who stated they would present to a hospital for stroke symptoms versus those who did not. Participants who preferred a hospital had a higher socioeconomic status score than other respondents (0.29 vs. 0.19, *p* < 0.001). There was otherwise no association between preference for a hospital and age, gender, urban residence, education level, ownership of health insurance, or tribal affiliation. Furthermore, there was no association between preference for hospital and conventional symptom knowledge or perception of self-risk.

## Discussion

In an area of East Africa with high prevalence of stroke, most residents said they would present to a hospital for stroke symptoms, which is an encouraging finding. However, community knowledge of stroke symptoms was poor, and perception of self-risk was very low. Preference for a hospital was associated with higher socioeconomic status, but not with knowledge of stroke symptoms or perception of self-risk. These findings suggest that knowledge of specific stroke symptoms may not be necessary to recognize that certain symptoms require hospital-level care, and they also suggest that economic considerations may be important drivers of health-care-seeking behavior for stroke.

Only 27% of respondents could name a conventional stroke symptom, and less than 1% were able to identify 3 symptoms. These proportions are lower than what has been reported outside of SSA using similarly open-ended questions: in studies from North America, Europe, and Asia, between 51 and 73% of laypeople could correctly identify at least one stroke symptom without a picklist [27–31]. Community knowledge of stroke symptoms in Tanzania was also lower than what has been reported in West Africa. In Nigeria, for example, 40% of laypersons could name a stroke symptom without a pick list [12]. For comparison, a table presenting the results of key studies regarding knowledge of stroke symptoms in SSA is included in the online supplementary material. Awareness of certain symptoms, such as trouble seeing, numbness, and dizziness, was especially poor in our study, with fewer than 3% of respondents identifying these symptoms. Respondents mentioned certain atypical symptoms such as fever or palpitations more often than they mentioned conventional symptoms such as numbness or trouble seeing. Thus, educational interventions to improve stroke knowledge in Tanzania should emphasize symptoms like numbness with low levels of community recognition.

Only 7.6% of adult respondents thought they had a chance of suffering a stroke, an alarmingly low proportion given the high local prevalence of risk factors and growing prevalence of disease [20, 22, 23]. To our knowledge, only one other study of perceptions of self-risk in SSA has been reported to date, but in that study, over half of adult Ugandans thought they had a chance of having a stroke [17]. The comparatively low levels of perceived self-risk in our study suggest that the need for education regarding the present and growing risk of stroke is particularly acute in Tanzania. Older age was not associated with perception of self-risk, and females were significantly less likely to consider themselves at risk of stroke in our study, suggesting that educational interventions should be targeted to elderly and female populations.

Despite low levels of symptom knowledge and self-perception of risk, a large majority of respondents stated they would present to a hospital for stroke-like symptoms. Preference for a hospital is important in Tanzania because other facilities typically do not have access to the necessary diagnostic modalities such as computed tomography or treatments such as thrombolytics and physical rehabilitation. The large proportion of participants who would present to a hospital for stroke symptoms is encouraging. However, it remains unknown whether prompt presentation to a hospital for stroke in SSA results in improved outcomes. Although early presentation and treatment in high-income countries are associated with better long-term neurologic outcomes [19], the benefit of presentation to a hospital is unknown in SSA, where delays in health-care seeking are common and use of thrombolytics for stroke is rare [32, 33].

Knowledge of symptoms and perception of self-risk were not associated with preference for a hospital for a scenario of stroke symptoms. The relationship between stroke symptom awareness and health-care-seeking behavior worldwide is heterogenous. Better symptom knowledge was associated with appropriate care seeking in a handful of studies performed in Asia [34–36], whereas most studies performed in Europe and North America have found no such association [37–40]. Our study also found no such association, suggesting that educational programming regarding stroke symptoms may performed in Europe and North America have found no such association [37–40]. Our study also found no such association, suggesting that educational programming regarding stroke symptoms may additional research is needed to aid in formulation of effective public health interventions to increase rapid hospital presentation for strokes. On bivariable analysis, the only predictor of preference for a hospital for stroke symptoms was higher socioeconomic status, although this association may be due to unmeasured confounders. This finding is consistent with the results of a study in Zimbabwe which found that patients with delayed hospital presentation for stroke reported more difficulty affording care [32]. Together, these findings suggest that reduction of financial barriers to stroke care in SSA might result in prompter presentation to hospitals.

This study had several limitations. First, only self-identified health-care decision makers were eligible for study participation, in order to survey those whose opinions drive actual health-care utilization. Thus, the study sample may not have been representative of Tanzanian adults as a whole. Furthermore, because this study was performed during daytime hours in the home, males and those with occupations outside the home were likely underrepresented in the study sample. Additionally, social desirability bias may have pushed some respondents to report that they would present to a hospital for stroke symptoms if they perceived this to be the most acceptable answer; therefore, the true proportion of adults presenting to a hospital for stroke may be lower than what is reported here. Moreover, participants were not asked to report how quickly they would present to their preferred facility for stroke symptoms. Collecting such information would have allowed for an assessment of presentation delays, another important consideration in stroke care-seeking behavior. Furthermore, we did not assess participants’ awareness of stroke causes or sources of knowledge regarding stroke in this study. Obtaining this information in future studies would be beneficial in the design of community educational interventions regarding stroke. Finally, information about individual respondents’ stroke risk profiles was not collected which would have allowed for a more nuanced analysis of determinants of stroke knowledge, perception of risk, and health-care seeking behavior. Nonetheless, given the known high local burden of risk factors such as hypertension, the low proportion of respondents in this study who knew a conventional stroke symptom or perceived themselves to be at risk of stroke, and the lack of association between age and stroke symptom knowledge or perception of self-risk in our study, there is clearly a need for community stroke education in northern Tanzania.

## Conclusions

Knowledge of stroke symptoms and perception of self-risk of stroke are low in northern Tanzania, but the majority of adults would present to a hospital for stroke-like symptoms. There is an urgent need for community education interventions regarding stroke in northern Tanzania, and additional research is needed to examine the barriers to prompt hospital-based stroke care in Tanzania and SSA at large.

## Supplementary Material

Supplemental

## Figures and Tables

**Table 1.** Sociodemographic features of household survey respondents, Northern Tanzania, 2018 (*n* = 670)

	Number of respondents (%)
Gender, female	469 (70.0)
Urban residence	154 (23.0)
Education	
None	27 (4.0)
Primary	494 (73.7)
Secondary	109 (16.3)
Post-secondary	40 (6.0)
Have health insurance	212 (31.6)
Religion	
Christian	547 (81.6)
Muslim	103 (15.4)
Other	20 (3.0)
Chagga tribe	506 (75.5)
Age, years, median (range)	47.0 (16−98)
Household size, number of persons, median (range)	3.0 (1−12)
SES score, median (range)	0.16 (0−1.01)

SES, socioeconomic status.

**Table 2.** Symptoms of stroke cited by residents of Northern Tanzania, 2018 (*n* = 670)

Symptom of stroke	Number of respondents (%)
Don’t know any	407 (60.7)
Focal weakness	135 (20.1)
Loss of consciousness	43 (6.4)
Confusion/trouble speaking	42 (6.3)
Generalized fatigue	29 (4.3)
Fever	20 (3.0)
Headache	18 (2.7)
Dizziness	17 (2.5)
Palpitations	15 (2.2)
Arm pain	10 (1.5)
Jaw pain	7 (1.0)
Numbness	7 (1.0)
Shortness of breath	3 (0.4)
Trouble seeing	1 (0.1)
Other	59 (8.8)

**Table 3.** Preferred health-care facility for stroke symptoms among residents of Northern Tanzania, 2018 (*n* = 670)

Facility	Number of respondents (%)
Hospital	558 (88.3)
Health center	57 (8.5)
Dispensary	49 (7.3)
Don’t know	3 (0.4)
Self-treatment at home	2 (0.3)
Traditional healer	1 (0.1)

**Table 4.** Characteristics of respondents who would present to hospital for stroke symptoms versus those who would not, Northern Tanzania, 2018 (*n* = 670)

	Hospital first choice for stroke symptoms (*n* = 558), *n* (%)	Hospital not first choice for stroke symptoms (*n* = 112), *n* (%)	OR (95% CI)	*p* value
Gender, female	395 (70.8)	74 (66.1)	1.25 (0.80−1.91)	0.320
Urban residence	126 (22.6)	28 (25.0)	0.87 (0.55−1.42)	0.579
Post-primary education	131 (23.5)	18 (16.1)	1.59 (0.95−2.82)	0.085
Has health insurance	176 (31.5)	36 (32.1)	0.97 (0.63−1.51)	0.901
Chagga tribe	428 (76.7)	78 (69.6)	1.44 (0.91−2.24)	0.113
Knows at least 1 conventional stroke symptom	160 (28.7)	24 (21.4)	1.47 (0.91−2.44)	0.117
Perceives self to be at risk of stroke	42 (7.5)	9 (8.0)	0.92 (0.45−2.08)	0.853
Age, years, mean (SD)	47.4 (18.9)	51.2 (20.3)		0.073
SES score, mean (SD)	0.29 (0.31)	0.19 (0.21)		<0.001*

* *p* < 0.05.

SES, socioeconomic status.
