# Supplementary material for: Perceptions of Stroke and Associated Health-Care-Seeking Behavior in Northern Tanzania: A Community-Based Study
Source: Neuroepidemiology. Author manuscript; Available in PMC 2020 Apr 15. (PMC6842572; doi:10.1159/000499069)
Supplement: Supplemental [file NIHMS1057419-supplement-Supplemental.pdf]

Perceptions of Stroke and Healthcare Seeking Behavior Questionnaire

Healthcare Utilization Survey

Northern Tanzania, 2018

[Swahili translation in brackets]

1. Please enter the surveyor's initials [Tafadhali weka herufi za mwanzo za majina ya mtafiti]:

2. Enter household ID [Ingiza namba ya kaya]:

3. District name [Jina la wilaya]:

4. Ward name [Jina la Kata]:

5. Village name [Jina la Kijiji]:

6. Are you the adult household member and decision-maker with respect to daily healthcare/healthcare utilization for the entire household and its members [Je wewe ni mtu mzima mwenye maamuzi katika familia hii katika masuala yanayohusu matumizi ya huduma za afya za kila siku kwa familia nzima]?

- ☐ Yes [Ndio]
- ☐ No [Hapana]

7. Provide the full name of the respondent [Andika majina kamili ya anayehojiwa]:

8. Provide the age (in YEARS) of the respondent [Umri wa anayehojiwa]:

9. Record the gender of the respondent [Jiinsia ya anaehojiwa].

- ☐ Male [Kiume]
- ☐ Female [Kike]

10. How many household members are living in this house in total (at the day of the visit) [Kuna jumla ya wakazi wangapi wanaoishi katika kaya hii]?

11. What are the symptoms or signs of a stroke [Zipi ni dalili au viashiria vya stroke/kiharusi]?
Instructions: List all. Surveyor is not to offer suggestions or show the screen to the respondent.

12. Do you think you have a chance of having a stroke [Je, unadhani kwamba una uwezekano wa kupata stroke/kiharusi]?

- [ ] Yes [Ndio]
- [ ] No [Hapana]
- [ ] Don’t know [Sijui]

13. Record the FIRST CHOICE healthcare facility where you or an adult household member would seek care if they developed sudden unilateral paralysis, trouble speaking, trouble walking, confusion, or vision problems [Andika chaguo la kwanza la kituo cha huduma za afya ambapo mkazi wa kaya hii (mwenye umri zaidi ya 17) angekwenda kutafuta huduma endapo angepata udaifu wa upande umoja, shida ya kuongea vizuri, shida ya kutembea vizuri, shida ya kuona vizuri, au kuchanganikiwa ghafla].
Instructions: Select one [Maelekezo: Chagua moja]

- [ ] Clinic [Kliniki]
- [ ] Dispensary [Zahanati]
- [ ] Health center [Kituo cha afya]
- [ ] Hospital [Hospitali]
- [ ] Pharmacy [Duka la dawa]
- [ ] Self treatment [Kumtibu au kujitibu nyumbani]
- [ ] Traditional healer [Mganga wa kienyeji]
- [ ] Watchful waiting [Kutofanya chochote, kumwangalia au kujiangalia nyumbani]
- [ ] Don’t know [Sijui]

14. If hospital selected, please specify the hospital [Tafadhali weka bayana hospitali].

- [ ] Huruma Hospital
- [ ] Kibosho Hospital
- [ ] Kilema Hospital
- [ ] KCMC
- [ ] Marangu Hospital
- [ ] Mawenzi Regional Hospital
- [ ] Moshi Arusha Hospital
- [ ] St. Joseph's Hospital
- [ ] TPC Hospital
- [ ] Other, specify: _______ [Nyingine, taja: ______]

15. Record the SECOND CHOICE healthcare facility where you or an adult household member would seek care if they developed sudden unilateral paralysis, trouble speaking, trouble walking, confusion, or vision problems [Andika chaguo la pili la kituo cha huduma za afya ambapo mkazi wa kaya hii (mwenye umri zaidi ya 17) angekwenda kutafuta huduma endapo angepata udaifu wa upande umoja, shida ya kuongea vizuri, shida ya kutembea vizuri, shida ya kuona vizuri, au kuchanganikiwa ghafla].
Instructions: Select one [Maelekezo: Chagua moja]

- [ ] Clinic [Kliniki]
- [ ] Dispensary [Zahanati]

- ☐ Health center [Kituo cha afya]
- ☐ Hospital [Hospitali]
- ☐ Pharmacy [Duka la dawa]
- ☐ Self treatment [Kumtibu au kujitibu nyumbani]
- ☐ Traditional healer [Mganga wa kienyeji]
- ☐ Watchful waiting [Kutofanya chochote, kumwangalia au kujiangalia nyumbani]
- ☐ Don’t know [Sijui]

16. If hospital selected, please specify the hospital [Tafadhali weka bayana hospitali].
- ☐ Huruma Hospital
- ☐ Kibosho Hospital
- ☐ Kilema Hospital
- ☐ KCMC
- ☐ Marangu Hospital
- ☐ Mawenzi Regional Hospital
- ☐ Moshi Arusha Hospital
- ☐ St. Joseph's Hospital
- ☐ TPC Hospital
- ☐ Other, specify: _______ [Nyingine, taja: ______]

17. What is the highest completed level of formal school education of the head of the household? [Mkuu wa kaya ana elimu ya kiwango gani]?
- ☐ No education
- ☐ Some primary school
- ☐ Completed primary school
- ☐ Some secondary school
- ☐ Completed secondary school
- ☐ Some college
- ☐ Completed college

18. What is the main occupation of the head of the household? [Mkuu wa kaya anafanya kazi gani]?
Instructions: Select one [Maelekezo: Chagua moja]
- ☐ Driver [Dereva]
- ☐ Guard/police [Polisi]
- ☐ Healthcare worker [Mhudumu wa afya]
- ☐ House girl [Msichana wa kazi]
- ☐ Housewife [Mama wa nyumbani]
- ☐ Livestock keeper [Mfugaji]
- ☐ Unskilled laborer [Wafanyakazi wasio na ujuzi wa kusomea]
- ☐ Street vendor [Machinga]
- ☐ Religious occupation [Mhudumu wa dini]

- [ ] Farmer [Mkulima]
- [ ] Student [Mwanafunzi]
- [ ] Teacher [Mwalimu]
- [ ] Tradesperson [Fundi]
- [ ] Unemployed [Hajaajiriwa]
- [ ] Office worker [Mfanyakazi wa ofisini]
- [ ] Small business manager [Mjasiriamali]
- [ ] Business Owner [Mfanyabishara]
- [ ] Other, specify: _______ [Nyingine, taja: ______]

19. What is the floor of your home made of [Je, sakafu ya nyumba hii imejengwa kwa kutumia malighafi gani]?
    - [ ] Brick [Tofali]
    - [ ] Cement/concrete [Simenti/zege]
    - [ ] Tiles [Vigae]
    - [ ] Mud [Tope]
    - [ ] Wood [Mbao]
    - [ ] Straw/leaves [Majan/matawi]
    - [ ] Metal/tin [Bati/debe]
    - [ ] Other, specify: _______ [Nyingine, taja: ______]
    - [ ] Don’t know [Sijui]

20. What is the wall of your home made of [Je, kuta za nyumba hii zimejengwa kwa kutumia malighafi gani]?
    - [ ] Brick [Tofali]
    - [ ] Cement/concrete [Simenti/zege]
    - [ ] Tiles [Vigae]
    - [ ] Mud [Tope]
    - [ ] Wood [Mbao]
    - [ ] Straw/leaves [Majan/matawi]
    - [ ] Metal/tin [Bati/debe]
    - [ ] Other, specify: _______ [Nyingine, taja: ______]
    - [ ] Don’t know [Sijui]

21. What is the roof of your home made of [Je, paa la nyumba hii limejengwa kwa kutumia malighafi gani]?
    - [ ] Brick [Tofali]
    - [ ] Cement/concrete [Simenti/zege]
    - [ ] Tiles [Vigae]
    - [ ] Mud [Tope]
    - [ ] Wood [Mbao]
    - [ ] Straw/leaves [Majan/matawi]

☐ Metal/tin [Bati/debe]
☐ Other, specify: _______ [Nyingine, taja: ______]
☐ Don’t know [Sijui]

22. Do you have access to electricity in your household [Je, kuna umeme kwenye nyumbani hii]?
☐ Yes [Ndio]
☐ No [Hapana]
☐ Don’t know [Sijui]

23. Which of the following items do members of this household own [Kipi kati ya vitu vifuatavyo kinamilikiwa na wakazi wa kaya hii]?
Instructions: Choose all that apply [Maelekezo: Chagua zote zinazohusika]
☐ Radio [Redio]
☐ Television [Televisheni]
☐ Mobile phone [Simu ya mkononi]
☐ Iron [Pasi]
☐ Refrigerator [Friji]
☐ Motorcycle [Pikipiki]
☐ Car or truck [Gari]
☐ Bank account [Akounti/Akiba ya benki]
☐ Tap water [Maji ya bomba]
☐ None of the above [Hakuna kati ya vilivyotajwa]

24. Does anyone in this household have any kind of health insurance [Je,wewe ama mkazi yeyote anahudumiwa na bima ya afya]?
☐ Yes [Ndio]
☐ No [Hapana]
☐ Don’t know [Sijui]

25. If yes, Which of the following health insurance plans are members of this household members enrolled in [Wanafamilia wana tumia aina gani ya bima ya afya]?
Instructions: Choose all that apply [Maelekezo: Chagua zote zinazohusika]
☐ National Health Insurance Fund (NHIF) [Mfuko wa taifa wa Bima ya Afya]
☐ Social Health Insurance Benefits (SHIB) [Mfuko wa Bima ya afya ya jamii]
☐ Community Health Fund (CHF) or Tiba Kwa Kadi (TIKA)
☐ Private insurance [Bima ya binafasi]
☐ Other, specify: _______ [Nyingine, taja: ______]
☐ Don’t know [Sijui]

26. Which religion(s) do members of this household belong to [Je, wakazi wa kaya hii wana amini katika dini ipi]?
☐ Christian [Ukristo]

- [ ] Muslim [Uislamu]
- [ ] Hindu [Hindu]
- [ ] None [Hakuna]
- [ ] Other, specify: _______ [Nyingine, taja: ______]

27. What tribes do members of this household belong to [Je,wakazi wa kaya hii ni kabila gani]?

- [ ] Chagga
- [ ] Pare
- [ ] Sambaa
- [ ] Masaai
- [ ] Meru
- [ ] Other, specify: _______ [Nyingine, taja: ______]

28. What type of toilet do members of this household usually use [Kwa kawaida ni aina gani ya choo ambacho hutumiwa na wakazi wa kaya hii]?

- [ ] Flush toilet [Choo cha maji (ndani ya nyumba)]
- [ ] Pit latrine [Choo cha shimo]
- [ ] Free range [Popote nje]
- [ ] Other, specify: _______ [Nyingine, taja: ______]

Supplementary Table

Supplementary Table 1. Summary of existing studies in sub-Saharan Africa reporting community knowledge of stroke symptoms

Author	Year	Country	Study population	Question format	Proportion aware of at least one stroke symptom
Wahab *et al* [1]	2008	Nigeria	Outpatients with hypertension and diabetes	Open-ended	39.6%
Akinyemi *et al* [2]	2009	Nigeria	Both clinical and non-clinical workers at a single hospital	Open-ended	91.4%
Cossi *et al* [3]	2012	Benin	Community members aged > 15 years	Open-ended	33.0%
Donkor *et al* [4]	2014	Ghana	Community members aged 18-60	Picklist	78.1%
Nakibuuka *et al* [5]	2014	Uganda	Adult community members	Open-ended	24.9%
Obembe *et al* [6]	2014	Nigeria	University students and staff	Picklist	87.8%
Kaddumukasa *et al* [7]	2015	Uganda	Community members aged > 18 years	Open-ended	43.0%
Komolafe *et al* [8]	2015	Nigeria	Students and teachers at secondary schools	Picklist	76.3%
Nansseu *et al* [9]	2017	Cameroon	Hospital patients or visitors aged > 18 years	Picklist	98.3%
Arisegi *et al* [10]	2018	Nigeria	Outpatients with hypertension and diabetes	Picklist	87.0%

References

1 Wahab KW, Okokhere PO, Ugheoke AJ, Oziegbe O, Asalu AF, Salami TA: Awareness of warning signs among suburban Nigerians at high risk for stroke is poor: a cross-sectional study. BMC neurology 2008;8:18.

2 Akinyemi RO, Ogah OS, Ogundipe RF, Oyesola OA, Oyadoke AA, Ogunlana MO, Otubogun FM, Odeyinka TF, Alabi BS, Akinyemi JO, Osinfade JK, Kalaria RN: Knowledge and perception of stroke amongst hospital workers in an African community. European journal of neurology 2009;16:998-1003.
3 Cossi MJ, Preux PM, Chabriat H, Gobron C, Houinato D: Knowledge of stroke among an urban population in Cotonou (Benin). Neuroepidemiology 2012;38:172-178.
4 Donkor ES, Owolabi MO, Bampoh P, Aspelund T, Gudnason V: Community awareness of stroke in Accra, Ghana. BMC public health 2014;14:196.
5 Nakibuuka J, Sajatovic M, Katabira E, Ddumba E, Byakika-Tusiime J, Furlan AJ: Knowledge and Perception of Stroke: A Population-Based Survey in Uganda. ISRN stroke 2014;2014
6 Obembe AO, Olaogun MO, Bamikole AA, Komolafe MA, Odetunde MO: Awareness of risk factors and warning signs of stroke in a Nigeria university. Journal of stroke and cerebrovascular diseases : the official journal of National Stroke Association 2014;23:749-758.
7 Kaddumukasa M, Kayima J, Kaddumukasa MN, Ddumba E, Mugenyi L, Pundik S, Furlan AJ, Sajatovic M, Katabira E: Knowledge, attitudes and perceptions of stroke: a cross-sectional survey in rural and urban Uganda. BMC research notes 2015;8:819.
8 Komolafe MA, Obembe AO, Olaogun MO, Adebiyi AM, Ugalahi T, Dada O, Kanu A, Adebiyi OC, Akilo F, Ogunkoya B, Fawale B: Awareness of stroke risk factors and warning signs in Nigerian adolescents compared with adults. Journal of stroke and cerebrovascular diseases : the official journal of National Stroke Association 2015;24:687-693.
9 Nansseu JR, Atangana CP, Petnga SN, Kamtchum-Tatuene J, Noubiap JJ: Assessment of the general public's knowledge of stroke: A cross-sectional study in Yaounde, Cameroon. Journal of the neurological sciences 2017;378:123-129.
10 Arisegi SA, Awosan KJ, Oche MO, Sabir AA, Ibrahim MT: Knowledge and practices related to stroke prevention among hypertensive and diabetic patients attending Specialist Hospital, Sokoto, Nigeria. The Pan African medical journal 2018;29:63.
